# Tertiary lymphoid structures in head and neck squamous cell carcinoma

**DOI:** 10.1016/j.tranon.2024.101949

**Published:** 2024-04-08

**Authors:** Jing Zhu, Hui Lu, Kongcheng Wang, Baorui Liu, Jing Yan

**Affiliations:** a Department of Oncology, Nanjing Drum Tower Hospital Clinical College of Nanjing Medical University, Nanjing, PR China; b Department of Oncology, Nanjing Drum Tower Hospital Clinical College of Nanjing University of Chinese Medicine, Nanjing, PR China; c Department of Oncology, Nanjing Drum Tower Hospital, Affiliated Hospital of Medical School, Nanjing University, Nanjing, PR China

**Keywords:** Tertiary lymphoid structure, Tumor immune microenvironment, Prognostic and predictive biomarker, Head and neck squamous cell carcinoma, Therapeutic induction

## Abstract

Head and neck squamous cell carcinoma (HNSCC) is the sixth most common malignancy worldwide. Smoking, drinking, and human papillomavirus (HPV) infection are the main risk factors. Early-stage patients can benefit from radical surgery, chemotherapy, and radiotherapy, but the prognosis of locally advanced, recurrent, or metastatic patients is poor. Programmed cell death receptor 1 (PD-1) inhibitor significantly prolongs the survival of these patients, but only about 20 % of the population can benefit significantly. Exploring effective predictive indicators of immunotherapy efficacy and new therapeutic targets is necessary. Tertiary lymphoid structure (TLS) is an ectopic lymphoid organ formed in non-lymphoid tissues, which usually occurs in chronic inflammation including autoimmune diseases, infectious diseases, and tumors. The structure and function of TLS are similar to those of secondary lymphoid organs. The existence of TLS is closely related to the favorable prognosis and immune response of patients. This article will review the formation, prognosis, and predictive value of TLS as well as inducing TLS neogenesis in HNSCC.

## Highlights

• The presence of TLS is associated with a favorable prognosis in patients with HNSCC.
• TLS can provide indicative significance for the efficacy of anti-tumor therapy in HNSCC patients.
• Inducing TLS production in tumors to increase anti-tumor immune response is considered an attractive treatment strategy for HNSCC.

## Introduction

Head and neck cancer is the sixth most common malignancy around the world, of which more than 90 % is head and neck squamous cell carcinoma (HNSCC) [1]. Smoking, alcohol abuse, and human papillomavirus (HPV) infection are the primary risk factors [2,3], besides, betel nut intake and disorder of oral microflora can also participate in the occurrence of HNSCC [4,5]. Most patients are locally advanced or advanced when diagnosed. For patients who cannot undergo radical resection, radiotherapy and platinum-based chemotherapy are the major treatment options [6], but the 5-year survival rate after standard treatment is only 50 % [7]. Radiotherapy combined with cetuximab improved the objective response rate (ORR) of patients with locally advanced HNSCC, but did not significantly prolong the survival of patients [8]. Clinical trials Keynote-048 and Checkmate141 established the status of programmed cell death 1 (PD-1) monoclonal antibodies in the treatment of head and neck cancer [9,10]. The efficacy of its single drug is even better than that of chemotherapy combined with cetuximab, but only about 20 % of patients can benefit significantly [11]. In addition, the course of immunotherapy is long, bringing a heavy economic burden to many patients, and immunotherapy may cause immune-related adverse events, some even fatal [12]. Therefore, screening meaningful prognostic indicators and exploring new therapeutic targets for HNSCC have become prominent issues.

Tertiary lymphoid structure (TLS), also known as tertiary lymphoid organs or ectopic lymphoid structures, is organized aggregates of immune cells that arise postnatally in nonlymphoid tissues [13]. TLS has been detected in many cancers and is related to the survival benefits of patients. TLS contains antigen-presenting cells such as dendritic cells (DCs), B cells, and follicular dendritic cells (FDCs), which can activate tumor-infiltrating lymphocytes (TILs) in the tumor microenvironment (TME) to initiate anti-tumor immune responses. In addition, some studies have shown that TLS can predict the efficacy of immune checkpoint blockade (ICB) independently of PD-L1 expression [14], [15], [16]. As stimulating antitumor immune responses is regarded as the most effective cancer therapy for producing long-term responses [17,18], TLS is considered to be the key factor of tumor local immunity [19]. Inducing TLS neogenesis in tumors to increase the antitumor immune response is considered to be an attractive therapeutic strategy. In some preclinical studies, chemokines, cytokines, and other factors including lymphotoxin α (LTα) [20], tumor necrosis factor-α and interleukin-2 [21] were found that could induce the formation of TLS in head and neck tumor-bearing mice. However, the understanding of the formation, mechanisms of antitumor immunity, and the method of inducing TLS in HNSCC is limited. In this article, we review the formation, prognosis, and predictive value of TLS as well as inducing TLS neogenesis in HNSCC.

### Composition, formation, and detection of TLS in HNSCC

TLS is the aggregation of lymphoid and stromal cells formed in non-secondary lymphoid organs under pathological conditions, which often occurs in autoimmune disease, infection, transplant rejection, and cancer [22]. The structure of TLS is similar to the secondary lymphoid organ. In HNSCC and the majority of solid tumors, TLS is mainly composed of CD3^+^*T* cells surrounding CD20^+^*B* cells and CD21^+^ FDCs [14,23]. The high endothelial venules (HEVs) and lymphatic vessels are located at the edge of TLS [23].

**Fig. 1:**
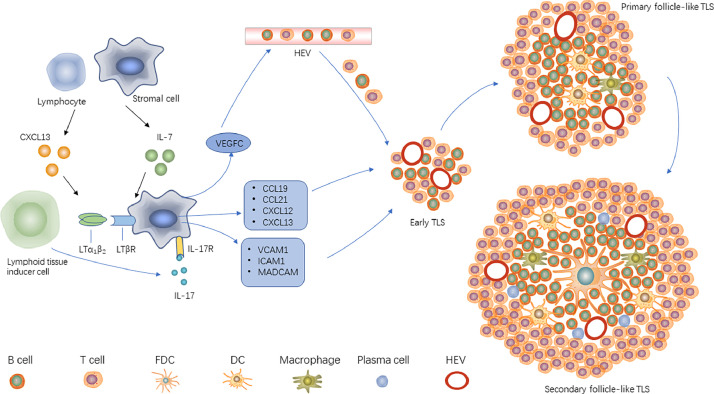
The formation mechanism of TLS. In the tumor microenvironment, lymphocytes or stromal cells in tumor tissue produce CXCL13 and IL-7 to recruit lymphoid tissue-induced cells under the stimulation of chronic inflammation. After a series of intercellular interactions, lymphocytes are recruited from the peripheral circulation through HEVs, ultimately promoting the formation of TLS and inhibiting tumor development. HEVs: high endothelial venules; ICAM1: intercellular adhesion molecule 1; LT: lymphotoxin; MAdCAM1: mucosal addressin cell adhesion molecule-1; VCAM1: vascular cell adhesion molecule 1; VEGFC: vascular endothelial growth factor-C.

Fig. 1 shows the formation process of TLS. Chronic inflammation induces lymphocytes or stromal cells to upregulate the expression of chemokines CXC ligand 13 (CXCL13) and interleukin 7 (IL-7), and the upregulated CXCL13 and IL-7 recruit lymphoid tissue inducer cells (LTi) such as Th17 cells, B cells, M1 macrophages to the site of inflammation [24,25]. Lymphotoxin-α1β2 (LTα1β2) expressed on the surface of LTi binds to LTβ receptor, IL-17, and IL-17 receptor on the surface of stromal cells. The combination of LTα1β2 and LTβR promotes the secretion of vascular endothelial growth factor C from stromal cells, induces the production of HEV and the secretion of adhesion molecules, such as vascular cell adhesion molecule 1 (VCAM-1), intercellular adhesion molecule 1 (ICAM-1) and mucosal addressin cell adhesion molecule-1 [26]. In addition, the cytokine IL-36γ secreted by macrophages and endothelial cells can increase the expression of VCAM-1 and ICAM-1 in stromal cells and vascular endothelial cells, and up-regulate the chemokines IL-8, CCL2, and CCL20 to enhance HEV ability to recruit lymphocytes, promoting TLS formation and maturation [27].

A recent study found that TGF-β could regulate the differentiation of T follicular helper cell (Tfh) cells and follicular regulatory T cells (Tfr) by regulating SATB1 (special AT-rich sequence-binding protein-1). Silencing SATB1 promoted the expression of Icos, thereby promoting Tfh cell differentiation. Tfh could promote TLS formation in tumors through the production of functional cytokines CXCL13/IL-21/LIGHT, activate B cell responses, and inhibit tumor growth [28]. Besides, the absence of endothelial notch signaling can promote the formation of TLS [29].

TLS maturation is a dynamic process and can be divided into two main categories according to the spatial structure and cellular composition. ①Early TLS: T cells and B cells gather irregularly, with only a small number of DCs between them, unable to generate an effective immune response. ②Mature TLS: A distinctive area of lymphocyte aggregation of T cells surrounded by B cells and a network of FDCs in the B cell area. Based on the presence or absence of germinal centers (GCs), mature TLS can be further classified into primary follicle-like TLS (without GC) and secondary follicle-like TLS (with GC). For the identification of the different maturation stages of TLS, characteristic molecular markers of immune cells can be detected using immunohistochemical/fluorescent staining techniques (Table 1) [30].

**Table 1:** Structural features and common markers of TLS at different stages of maturation.

Maturity stage	Structure characteristics	Identifying features	Common biomarkers
Early TLS	T cells and B cells mixed and irregularly distributed	No FDC networks and GCs	B cell: CD20/CD19 Naive T Cell: CD3 Cytotoxic T cell: CD3/CD8 Effector T cell: CD3/CD4 Regulatory T cell: FOXP3 Mature DC: LAMP3 (CD208)/CD83 FDC: CD21/CD23 GC B cells: CD21/CD23/Ki67/BVL-6/AID HEV: PNAd/MECA-79 Plasma Cell: CD138 Macrophage: CD68/CD163
Primary follicle-like TLS	B cells form a follicular structure at the center, surrounded peripherally by T cells and a small number of mature DCs	With FDC networks but without GCs	
Secondary follicle-like TLS	The appearance of lymph node-like GCs in follicular structures	With FDC networks and GCs	

There is significant heterogeneity in TLS among different solid tumors. High expression of TLS-related chemokines was observed in lung squamous cell carcinoma, lung adenocarcinoma, gastric adenocarcinoma, and HNSCC, while extremely low expression of TLS markers was observed in low-grade glioma and adrenal cortical carcinoma. Lechner et al. detected TLS at the invasive edge of HNSCC and proved that Tfh was a key participant in TLS, and the frequency of Tfh in TME was significantly increased in patients with detectable tumor-associated antigen response. This indicated that TLS played an important role in coordinating the anti-tumor efficacy and inducing the humoral immune response of HNSCC [31]. Gkegka et al. found that the density of TLS invading the front edge and internal tumor area was significantly reduced and was related to the high tumor budding and the increased expression of HIF1α and LDH5 in laryngeal squamous cell carcinoma (LSCC) [32]. Liang et al. found that the chemokine XCL2 produced by NK cells stimulates DC recruitment into the TME and XCL2 may be involved in TLS maturation and invasion in LSCC [33]. These studies are exciting, but there is still insufficient evidence on the molecular mechanisms underlying TLS formation, which deserves further exploration.

The methods for detecting TLS mainly include hematoxylin and eosin (H&E) staining, immunohistochemistry (IHC), and gene signatures based on RNA-seq. IHC, H&E, gene signatures based on RNA-seq and flow cytometry for detecting TLS had been used in some HNSCC studies.

### Prognostic and predictive potential of TLS in HNSCC

Favorable impact of TLS on prognosis in HNSCC.

Accumulating evidence indicates that TLS is usually associated with a good prognosis for most cancers (Table 2) [15,[34], [35], [36], [37], [38], [39], [40], [41], [42], [43], [44], [45]]. There have been many studies in HNSCC exploring the correlation between TLS and survival benefits, as summarized in Table 3. Wang et al. analyzed the TCGA database and performed immunohistochemical staining on 188 HNSCC patient specimens. The results indicated that patients with TLS with high infiltration of CD8+ cells had the best prognosis [20]. Liu et al. proposed to label TLS with 13 chemokines (LAMP3, CCL2, CCL3, CCL4, CCL5, CCL18, CCL19, CCL21, CXCL9, CXCL10, CXCL11, CXCK13, CXCR4) and divided the HNSCC samples of TCGA into TLS-hi and TLS-low groups. The TLS-hi group showed better overall survival (OS) [46].

**Table 2:** The values of TLS in the prognosis of various malignancies.

Cancer type	Number of cases	Main finding	Prognostic value	Reference
Non-small cell lung cancer	121	Patients with highly matured and dense TLS show improved DFS	Positive	Sun, [34]
Breast cancer	14	The presence of TLS is associated with prolonged DFS	Positive	Wang, [35]
Colorectal cancer liver metastases	603	Intra-tumour area TLS scores (T scores) are positively associated with prognosis, whereas peri‑tumour area TLS scores (P scores) are negatively associated with prognosis	T score: positive P score: negative	Zhang, [36]
Gastric cancer	292	Longer survival and better response to immunotherapy were seen in patients with higher TLS scores	Positive	Jiang, [37]
Pancreatic ductal adenocarcinoma	162	TLS is linked to prolonged cancer-specific survival	Positive	Tanaka, [38]
Hepatocellular carcinoma	273	Intratumor TLS in postoperative patients is significantly associated with a lower risk of early recurrence	Positive	Calderaro, [39]
Hepatocellular carcinoma	82	TLS in non-tumour liver tissue is associated with an increased risk of late recurrence and reduced OS after surgery	Negative	Finkin, [40]
Clear cell renal cell carcinoma	24	B cells in TLS gradually transform into plasma cells, and TLS is associated with effective response to immunotherapy	Positive	Meylan, 2022[41]
Bladder cancer	100	The TLS marker CXCL13 is associated with prolonged survival in advanced patients treated with immune checkpoint inhibitors	Positive	Groeneveld, [42]
Cervical cancer	93	TLS formation is associated with a better prognosis	Positive	Zhang, [43]
Ovarian cancer	570	TLS exerts anti-tumour effects	Positive	Kroeger, [44]
Melanoma	164	TLS can be a marker of a strong immune response and is positively associated with patient prognosis	Positive	Cabrita, [15]
Soft tissue sarcoma	30	TLS-positive patients have longer progression-free survival	Positive	Italiano, [45]

**Table 3:** **Prognostic and predictive potential of TLS in HNSCC**.

Tumor Type	Number of Patients	Identification Method and Markers	Characteristic of TLS	Potential Value	Impact Outcome	Reference
HNSCC	188	IHC, TLS markers (PNAd, CD20, and CD3)	Mature TLS was observed in 30 cases and immature TLS in 106 cases. Immature TLSs usually meanwhile developed when mature TLS existed. Patients with TLS vs without TLS. High TLS score vs medium TLS score vs low TLS score	Prognostic	OS, DFS	Wang, [20]
HNSCC	608	IHC, label TLS with 13 chemokines (LAMP3, CCL2, CCL3, CCL4, CCL5, CCL18, CCL19, CCL21, CXCL9, CXCL10, CXCL11, CXCK13, CXCR4)	TLS-hi vs TLS-low groups	Prognostic	OS	Liu, [46]
oral cancer	65	HE, TLS (dense lymphocytic clusters, the locations and counts of TLSs)	Peritumoral TLS: 75.4 % (49/65), intra-tumoural:33.8 % (22/65) Oral cancer-associated TLSs were classified into five categories: grade 0–4	Prognostic	DFS, OS	Li, [47]
OSCC	168	IHC, related markers of TLS (PNAd, CD20, and CD3).	26.8 % (45/168) was TLS positive Mature TLS: 10.1 % (17/168), immature TLS: 16.7 % (28/168) 94.1 % of mature TLS (16/17) are located in the stroma of the tumor margin, while 46.4 % of immature TLS (13/28) are located within the tumor	Prognostic	5-year OS, 5-year RFS	Li, [48]
OSCC	80	IHC, TLS (B-cells, FDCs, T-cells, GC B-cells, and HEVs).	21.3 % (17/80) was tumor-related TLS, mainly in the peritumoral stroma	Prognostic	5-year DSS	Wirsing, [50]
OTSCC	97	HE,IHC, TLS (early TLS, primary follicle-like TLS, and secondary follicle-like TLS).	The incidence of TLS was 76.3 % (74/97)	Prognostic	OS, DFS	Wang, [51]
OTSCC	310	HE, TLS (clusters of lymphocytes)	84.8 % (263/310) showed TLS around the tumor	Prognostic	DSS, OS	Almangush, [52]
OSCC	106	IHC, TLS (TCF7, CD3, CD20, and CD21)	32.1 % (34/106) was TLS-positive samples	Prognostic	PFS, OS	Peng, [53]
LSCC	70	HE, TLS/The expressions of PD-L1/TIL	Patients with TLS vs without TLS	Prognostic	DFS, RR	Alessandrini, [54]
LSCC	24	HE, TLS/The expressions of PD-L1/TIL	a higher CPS, a higher TIL count, and the presence of TLS	Prognostic	DFS, RR	Franz, [55]
HNSCC	496	RNA-seq data from TCGA, TLS (expression of gene signatures for B cells, naive B cells, memory B cells, and CD4+ *T* Cells).	Immunotype D is rich in TLS In the mouse model, the induction of TLS formation enhances the response to PD-1 inhibitors	Prognostic, predictive response	OS, CR	Li, [65]
HNSCC	38	Flow cytometry, TLS (T follicular helper cells (CD4+/CXCR5+/CD45RA-/CCR7-))	TLS mainly at the invasive margin TLS in different stages of development	Predictive response	The fraction of CD3+ cells and PD-1+ /ICOS+ Tfh	Lechner, [31]
LSCC	33	HE, TLS score (The sum of all TLSs was divided by the entire number of optical fields)	Two groups of low- and high-TLS density	Predictive response	Immune response	Gkegka,[32]

CR: complete response, DFS: disease-free survival, HE: hematoxylin-eosin staining, IHC: immunohistochemistry, LSCC: laryngeal squamous cell carcinoma, TLS: tertiary lymphoid structures, HNSCC: head and neck squamous cell carcinoma, OS: overall survival, OSCC: oral squamous cell carcinoma, OTSCC: oral tongue squamous cell carcinoma, RFS: recurrence-free survival, RR: recurrence rate, TCGA: The Cancer Genome Atlas.

In 65 patients with oral cancer, intratumoral and peritumoral TLS were analyzed, and the positive rates were 33.8 % and 75.4 %, respectively. Patients with higher TLS grades were bound up with better disease-free survival (DFS) and OS [47]. Li et al. detected the related markers of TLS (PNAd, CD20, and CD3) in 168 patients with oral squamous cell carcinoma (OSCC), and identified 45 patients (26.8 %) to be TLS-positive. Compared with TLS-negative patients, TLS-positive patients had improved 5-year OS and relapse-free survival rates. In addition, CD8^+^*T* cells and CD57^+^NK cells' density was higher in TLS-positive tissues. The combination of TLS, CD8^+^*T* cells, and CD57^+^NK cells could well predict the 5-year OS (AUC=0.730) [48]. Additionally, the existence of HEVs in TLS is associated with a favorable TME and is an independent positive prognostic marker of OSCC [49]. Immunohistochemical staining was used to examine the tumor tissues from 80 OSCC patients, and tumor-related TLS was found in 17 cases (21 %). The 5-year disease-specific survival (DSS) rate of TLS-positive patients was higher than that of negative patients (88.2% vs 60.3 %). Most TLS were found in highly differentiated and early clinical stage tissues, indicating that the formation of TLS may occur in the early stage of tumorigenesis [50]. Wang et al. reported that in 97 cases of the early clinical stage (cT1/2N0) oral tongue squamous cell carcinoma (OTSCC), the incidence of TLS was 76.3 %. The existence of TLS was beneficial to the prognosis and can be used as an independent prognostic factor for OS and DFS [51]. The tissue sections of 310 patients with early-stage (cT1–2N0) OTSCC from 5 centers were evaluated by hematoxylin-eosin staining, and 263 patients (84.8 %) showed TLS around the tumor (the invasive front area). The DSS and OS of these patients were significantly higher than those of patients without TLS. Besides, only 105 cases (33.9 %) found TLS in the tumor matrix, and these TLS were not related to survival [52]. Transcription factor 1 encoded by TCF7 participates in the Wnt signaling pathway and plays a role in T cell differentiation. By using single-cell profiling to capture the characteristics of OSCC cells, it was found that the TCF1/TCF7^+^*T* cell subgroup had high expressions of TLS-related genes (CCR7, SELL) and was positively correlated with TLS. The existence of TLS and the high frequency of TCF7^+^*T* cells were independent prognostic factors for improving OS [53].

The expressions of PD-L1, TIL, and TLS in 70 LSCC patients were studied by univariate/multivariate analysis. The recurrence rate of patients with TLS in tumor samples was significantly lower than those without TLS (5.26% vs 41.2 %), and DFS was also significantly prolonged. The presence of TLS was significantly correlated with the PD-L1 combined positive score ≥ 1 [54]. An Italian study evaluated the expression of PD-L1, TIL, and TLS in 24 LSCC patients who received postoperative radiotherapy and reached similar conclusions [55].

The above research results suggest that TLS is a prominent marker reflecting the state of TME. The composition, quantity, density, and localization of TLS in tumors could have an impact on patient prognosis [56]. In intrahepatic cholangiocarcinoma, Ding et al. quantified the TLS inside and outside the tumor, established the T score of the intratumoral region and the P score of the peritumoral region, and divided intrahepatic cholangiocarcinoma into four immune categories. The four categories indicated significantly different prognoses [57]. In resectable non-small cell lung cancer, TLS maturity was an independent prognostic factor for DFS in the neoadjuvant chemotherapy-immunotherapy group and the initial treatment group [58]. The maturity of TLS also affects the prognosis of LSCC. Liang et al. used HE staining to classify TLS maturity and found that three types of TLS had higher infiltration in the extratumoral area. Compared with the non-follicle-like (FL) TLS group, the FL-TLS group had more abundant immune cell infiltration and could be identified as an independent prognostic factor for LSCC [33]. In addition, HPV infection was reported to be significantly associated with high expression of TLS in HNSCC [59]. Compared with HPV-HNSCC patients, GC tumor-infiltrating B cells (TIL-B) and TLS with GC increase, are associated with a more favorable prognosis in HPV+HNSCC patients [60].

TLS functions in immunotherapy response in HNSCC.

TLS is an acquired immune structure that appears in non-lymphoid tissues and is considered to be the key factor of tumor local immunity [19]. TLS contains antigen-presenting cells such as DCs, B cells, and FDCs, which can activate TILs in TME to initiate anti-tumor immune responses. In addition, B cells instruct T cells to recognize tumor-associated antigens and enhance local immune response near TLS [61,62]. In a study of metastatic melanoma, it was found that TLS could promote T-cell differentiation [15], and HEVs in TLS were positively correlated with lymphocyte infiltration [63]. In the mouse model of colon cancer, intravenous injection of GFP splenocytes leads to the homing of lymphocytes to TLS in colon mucosa, which indicates that TLS plays an active role in recruiting lymphocytes to tumor areas [64].

Previous studies have shown that TLS can predict the efficacy of ICB independently of PD-L1 expression [14], [15], [16]. The study of 136 melanoma patients treated with ICB combined with neoadjuvant chemotherapy found that the density of CD20^+^*B* cells in the response group was significantly higher than that in the non-response group, and these B cells were located in TLS [14]. Italiano et al. explored the efficacy of pembrolizumab in advanced sarcoma patients with TLS and found that the ORR and PFS in patients with TLS were significantly better than those patients without TLS [45]. These findings suggest that TLS can help screen patients who can benefit from immunotherapy.

Several studies in HNSCC have also revealed the correlation between TLS and treatment response, as listed in Table 3. The immune classification of TME in HNSCC was demonstrated by analyzing the TCGA dataset, and it was found that immunotype D rich in TLS had a better response to ICB treatment. A mouse model of HPV-HNSCC rich in TLS was established by overexpressing LIGHT in the mouse HNSCC cell line. In this mouse model, the induction of TLS formation enhanced the response to PD-1 inhibitors [65]. Lechner et al. detected TLS at the invasive edge of HNSCC proved that Tfh was a key participant in TLS, and found that the frequency of Tfh in TME was significantly increased in patients with detectable tumor-associated antigen response. This indicated that TLS played an important role in coordinating the anti-tumor efficacy and inducing the humoral immune response of HNSCC [31]. A study analyzed the expression of TLS in 33 patients with LSCC who underwent laryngectomy. The results showed that the density of TLS invading the front edge and internal tumor area was significantly reduced, and was related to the high tumor budding and the increased expression of HIF1α and LDH5 [32].

In general, distinguishing the composition, distribution, and function of cell subtypes within TLS will help us clearly distinguish the components that inhibit tumor progression and promote tumor progression within TLS, as well as their comprehensive functional effects, and provide the theoretical basis for tumor immunotherapy.

### Therapeutic induction of TLS in HNSCC

At present, studies have confirmed that tumor tissue with mature TLS has a high response to immunotherapy, so inducing the production of mature TLS has become a direction of anti-tumor therapy. A series of methods are currently being developed, such as the use of chemokines, cytokines, antibodies, antigen-presenting cells, etc., to promote the formation of TLS in tumors and promote tumor immunity [66].

Using a mouse model of mesothelioma, Liu et al. demonstrated that the immunotoxin LMB-100 could recruit TILs into tumors and induce the formation of TLS, enhancing the antitumor immune response [67]. In an orthotopic murine model of pancreatic ductal adenocarcinoma, intratumoral injection of the lymphoid chemokine CXCL13/CCL21 induced TLS formation and improved tumor response to systemic chemotherapy with gemcitabine, which was associated with TLS-associated B cell-mediated DC maturation [68]. Intraperitoneal injection of recombinant CXCL13 into ovarian cancer mice induced TLS formation in tumors, which in turn promoted CD8-positive T cells in the TME and prolonged survival [69]. Regulating the flora may induce the formation of TLS. When introduced Helicobacter pylori into the mouse model of colorectal cancer, the number of T follicular helper cells in the colon increased, and TLS was induced to maturation [70].

In HNSCC, existing articles have identified some common TLS-promoting cytokines and chemokines such as CXCL13, which may be exploited to promote lymphoblastogenesis, lymphocyte recruitment, and DC homing, increase TLS formation and Inhibit regulatory T cells, and finally achieve the purpose of anti-tumor. In LSCC, the chemokine XCL2 produced by NK cells stimulates DC recruitment into the TME, suggesting its possible involvement in TLS maturation and invasion [33]. So, inducing the generation of TLS in tumor tissue may be a promising direction for immunotherapy in HNSCC patients. Clubb et al. developed an adenovirus encoding tumor necrosis factor-α and interleukin-2 (Ad5/3-E2F-D24-hTNFa-IRES hIL-2) that could induce the formation of TLS, enhance ICB efficacy, inhibit tumor growth, and prolong survival in tumor-bearing mice with refractory head and neck cancer [21]. LTα was overexpressed in SCC7 cells, and a mouse tongue cancer model was established. The aggregation of T cells in the LTα cell group increased, and TLS gradually formed. In addition, the tumor volume in Group LTα was significantly smaller than that in the control group, and the number of infiltrating CD8^+^*T* cells increased [20]. Wen et al. used tannic acid to interact with CpG/Mn2+/Epstein-Barr virus nuclear antigen 1 to synthesize a double-adjuvanted nanovaccine pECM. The nanovaccine could activate LT-α and LT-β pathways, and subsequently enhance the expression of downstream chemokines CCL19/CCL21, CXCL10, and CXCL13 in the TME, thereby promoting the formation of TLS, significantly enhancing the local immune response, and finally inhibiting the mimicry nasopharyngeal carcinoma development [71].

Existing treatment strategies such as surgery, radiotherapy, and chemotherapy may also affect the TME and lead to changes in TLS. Sun et al. analyzed TLS in tumor tissues of three cohorts (neoadjuvant chemotherapy immunotherapy group, neoadjuvant chemotherapy group, and initial treatment group) of patients with resected stage II-IIIA non-small cell lung cancer (NSCLC), suggesting that PD-1 inhibitors might promote TLS maturation, while chemotherapy could inhibit TLS maturation [58]. Similar results have been found in HNSCC. The mRNA expressions of 730 immune-related genes were analyzed in 18 patients with HNSCC receiving radiotherapy and chemotherapy (CRT). CCL19, CCR7, CXCL13, CXCR5, CCL14, and LTB were reported to participate in the formation of TLS. After adjuvant CRT, the expressions of immune-related genes were generally reduced, and the number of TLS was significantly reduced. This decline was not obvious in postoperative recurrent tumors without chemoradiotherapy [72].

### Conclusion and perspectives

Recent studies describe that TLS can recruit lymphocytes and generate an effective anti-tumor immune response locally. Many studies have shown that TLS is observed in HNSCC, and most of them are positively correlated with patients' good prognosis and immunotherapy response. In a preclinical study, an adenovirus encoding tumor necrosis factor-α and interleukin-2 was found that induce the formation of TLS, enhance ICB efficacy, inhibit tumor growth, and prolong survival in head and neck tumor-bearing mice [24]. Furthermore, in mouse models, the possibility of local TLS induction via tissue-specific expression of TLS-associated cytokines and chemokines has been established [13]. Therefore, developing treatment methods to induce TLS may be a promising treatment for HNSCC.

Although many studies have suggested that the presence of TLS is associated with a better prognosis in HNSCC, studies on the role of the maturity and spatial distribution of TLS in the prognosis of patients are still lacking. In addition, the prognosis of HNSCC patients in different parts is not the same, and it may be more appropriate to explore the prognostic effect of TLS by disease type. Most of the current studies are retrospective studies, the number of patients is small, and large-scale multi-center prospective studies are still lacking. Future methods for assessing TLS need to be standardized to facilitate robust detection and quantification of TLS in tumor samples. In addition, a lot of research is still needed to explore the formation mechanism of TLS and the methods to induce TLS formation in HNSCC. Perhaps these will be future research directions.

## CRediT authorship contribution statement

**Jing Zhu:** Writing – original draft. **Hui Lu:** Writing – original draft. **Kongcheng Wang:** Writing – review & editing. **Baorui Liu:** Writing – review & editing. **Jing Yan:** Writing – review & editing.

## Declaration of competing interest

The authors declare that they have no known competing financial interests or personal relationships that could have appeared to influence the work reported in this paper.
